# Differences in Acaricide Susceptibility Between Parasitic Stages of *Rhipicephalus microplus*

**DOI:** 10.3390/pathogens15090968

**Published:** 2026-09-11

**Authors:** Daniel Sobreira Rodrigues, Ricardo Nascimento Araujo, Luccas Gabriel Ferreira Malta, Lorena Lopes Ferreira, Romário Cerqueira Leite

**Affiliations:** 1 Campo Experimental Santa Rita, Empresa de Pesquisa Agropecuária de Minas Gerais—EPAMIG Centro Oeste, Rodovia MG 424, KM 64, Prudente de Morais 35701-970, MG, Brazil; 2 Departamento de Parasitologia, Instituto de Ciências Biológicas—ICB, Universidade Federal de Minas Gerais—UFMG, Av. Pres. Antônio Carlos, 6627, Pampulha, Belo Horizonte 31270-901, MG, Brazil; rnaraujo@icb.ufmg.br (R.N.A.); luccasmalta10@hotmail.com (L.G.F.M.); 3 Departamento de Medicina Veterinária Preventiva, Escola de Veterinária-EV, Universidade Federal de Minas Gerais—UFMG, Av. Pres. Antônio Carlos, 6627, Pampulha, Belo Horizonte 31270-901, MG, Brazil; lorenalopesf@gmail.com (L.L.F.); romarioleite@gmail.com (R.C.L.)

**Keywords:** *Rhipicephalus microplus*, cattle tick, metanymph, epicuticle, acaricide susceptibility, engorgement, resistance, field efficacy

## Abstract

Acaricide resistance is a major challenge for the control of *Rhipicephalus microplus*, and differences between laboratory and field efficacy may complicate the assessment of resistance. However, the influence of parasitic developmental stages on acaricide susceptibility remains poorly understood. Metanymphs were historically considered an acaricide-resistant stage, although this assumption was later questioned. To re-evaluate this issue, three experiments were conducted. First, replicated field spray treatments were performed to evaluate mortality over time. Second, feeding chambers attached to cattle were used to expose ticks at different stages to acaricide. Third, partially engorged females were sprayed and surviving engorged females were evaluated for reproductive performance. Field mortality varied according to the developmental stages corresponding to each assessment day. In feeding chambers, mortality reached 83% in non-engorged nymphs and 90% in young adults, compared with 29% in engorged nymphs and 8% in controls. Females surviving treatment while partially engorged maintained high reproductive performance, whereas subsequent acaricide immersion markedly reduced hatchability and egg production. These findings demonstrate stage-dependent differences in acaricide susceptibility and indicate that developmental and engorgement status may contribute to discrepancies between laboratory and field efficacy tests.

## 1. Introduction

The cattle tick, *Rhipicephalus microplus* (Canestrini, 1888), poses a major challenge to cattle production, particularly because the development of acaricide resistance can compromise the effectiveness of tick control. Therefore, laboratory and field tests are essential for assessing acaricide efficacy and detecting resistance in tick populations. In addition to resistance at the population level, differences in susceptibility among the parasitic stages of *R. microplus* have also been reported. The occurrence of parasitic stages of *R. microplus* more resistant than others, when subjected to the same acaricidal treatments, was suggested several times during the 1950s and 1970s [1,2,3,4]. At that time, there was a common understanding that metalarvae and metanymphs were “resistant forms” (metalarva and metanymph are transitional pre-molting phases between the larval–nymphal and nymphal–adult stages, respectively). The main hypothesis was that the maintenance of the epicuticule, together with the production of a new integument, would form a more efficient physical barrier against the absorption of contact acaricides. However, this concept was subsequently challenged by Grillo-Torrado and Gutiérrez [5], who questioned the presumed lower susceptibility of metanymphs to organophosphate acaricides. Following their findings, the hypothesis received little further attention in scientific literature. The authors made the following consideration on the concept: “En lo que atañe a causas externas y cuando las mismas están dadas por la actuación de garrapaticidas organofosforados sobre las distintas formas biológicas del ácaro, esse concepto queda limitado o enteramente eliminado” (Regarding external causes, when these result from the action of organophosphate acaricides on the different biological forms of the mite, this concept becomes limited or is entirely eliminated).

In 1980, Souza [6] also demonstrated that ethion (0.12%) and amitraz (0.025%) immersion were not lethal to metanymphs and partially engorged females from an organophosphate-resistant strain. However, unfed larvae, metalarvae, early female and male stages and fully engorged females showed distinct mortality values. Despite this, the issue remained in low evidence.

A few years ago, during a doctoral experiment to evaluate acaricide spray treatments with pyrethroid and organophosphorus combinations, high mortality values were observed in the dates corresponding to the stages of larvae to non-engorged nymphs, as well as to early female and male stages, when compared to the low mortalities in the dates corresponding to engorged nymphs, metanymphs, and partially and fully engorged females [7]. From these findings, in addition to the information available in the literature [1,2,3,4,6], it was possible to elaborate the hypothesis in which different parasitic stages have different levels of susceptibility when submitted to organophosphate and/or pyrethroid treatment using spray baths.

Despite these previous observations, differences in acaricide susceptibility among the parasitic developmental stages of *R. microplus* remain poorly understood and have received little attention in recent decades. Understanding whether some stages are less susceptible than others may help advance the knowledge of resistance mechanisms, identify critical points in tick control programs, and explain discrepancies between laboratory tests and field treatments. Therefore, the aim of this study was to assess differences in acaricide susceptibility among parasitic stages of *R. microplus* following spray treatments.

## 2. Materials and Methods

### 2.1. Experimental Design and Acaricide Formulations

The tests were conducted in an experimental herd with 3/4 Holstein × Zebu animals and in the Laboratory of Veterinary Parasitology of the Santa Rita Experimental Field, EPAMIG—Centro Oeste, located in Prudente de Morais, Minas Gerais (19°28′55″ S; 44°09′18″ W), Brazil. Three experiments were carried out to address different aspects of the same hypothesis.

In the first experiment, acaricides were sprayed on cattle under field conditions and the mortality values of *R. microplus* were assessed at different dates after the treatments. In the second, experimental tick infestations were carried out in feeding chambers, with the objective of evaluating, through photographic records, the mortality of individuals of different stages submitted to acaricide treatments. For the third experiment, experimental tick infestations were also conducted in feeding chambers, but treatments were performed on the phase of partially engorged females. The objective was to evaluate the viability of the individuals who survived the treatments. For this, fully engorged females were collected from the infestations, divided in groups, and submitted to the adult immersion tests (AITs) in comparison to the control group [8].

The acaricide treatment formulation used for the experiments was produced from a commercial product containing 15% cypermethrin and 25% chlorpyrifos, diluted in water to 0.02% and 0.033%, respectively. The product was chosen considering previously performed AITs prior to each experiment [8].

### 2.2. Experiment 1: Acaricide Treatment in Naturally Infested Cattle

Thirty-two dairy cows were used, distributed into four groups. The animals were kept in the same palisade grass (*Urochloa brizantha*) pasture areas, under similar conditions of parasitic challenge, for 51 days before and 21 days after the acaricide treatment. After sharing the same area for 30 days, three tick counts were performed at 1-week intervals. To achieve this, the animals were individually restrained on a trunk and inspected for *R. microplus* females with size ≥ 4 mm [9]. The procedure was performed at 06:00 a.m. on the right side of each animal, and the observed value was multiplied by two, according to [10,11]. The animals were classified in increasing order of parasite load and distributed in four groups with similar susceptibility profile and parasite load [12].

Three groups were treated with acaricide spray and were considered repetitions, and the control group was sprayed first with water. The spray treatments were performed on the animal’s entire body in sequence, by the same operator, using a power sprayer. Each animal received 1 L/100 kg of acaricide emulsion, and the control group was kept separate for 24 h before joining the others.

Tick counts were performed on days D + 1, D + 4, D + 8, D + 11, D + 14, D + 17, D + 19 and D + 21 after treatment. The acaricidal efficiency was calculated according to [10,11] and recommended by [13], by using the formula:

[(C − T)/C] × 100,

where:

C = number of ticks in the control group;

T = number of ticks in the acaricide-treated groups.

### 2.3. Experiment 2: Acaricide Treatment in Feeding Chambers

Two lactating cows were used and eight chambers to feed ticks were confectioned. The chambers had a circular shape and were made of leather, measuring 4 cm in internal diameter and 9 cm in external diameter, with fabric covering them to allow air entrance. In addition, there was a zipper to enable the inspection of the interior. Each chamber was fixed to the skin of the hosts by means of contact glue and four subcutaneous stitches. Before the procedure, the region was anesthetized by the epidural intercoccygeal spinal injection of 8 mL of 2% lidocaine, associated with 0.002% epinephrine, according to the technique described by [14].

**Figure 1:**
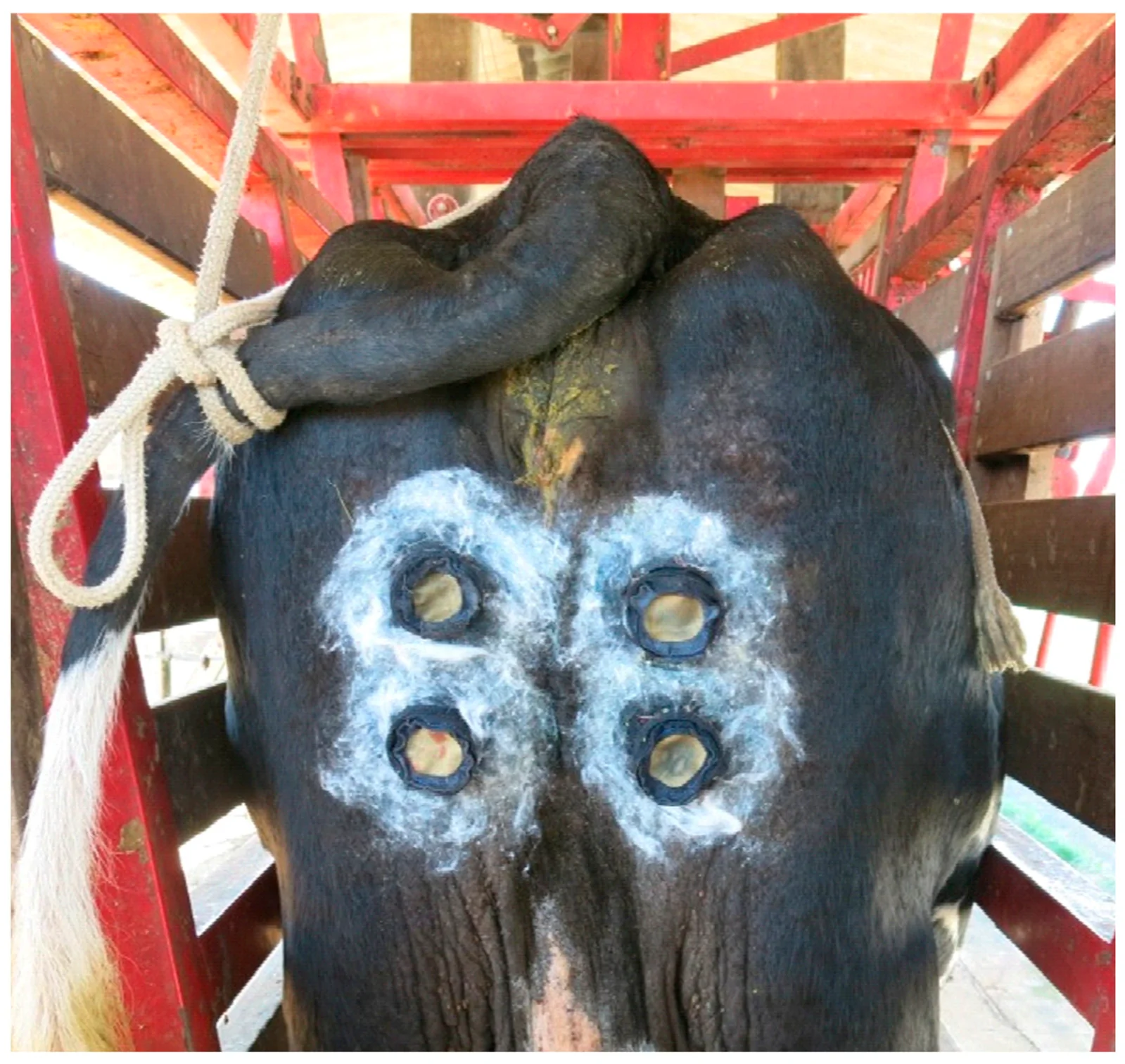
Tick feeding chambers implanted in cattle for conducting experimental infestations with *R. microplus* larvae.

In each cow, four chambers were fixed on the posterior region, located below the vulva and above the insertion of the posterior udder, in which experimental tick infestations were performed with 600 60-day-old larvae (Figure 1). Each chamber corresponded to one treatment: one control and three acaricide treatments carried out with the same emulsion depending on the day after tick infestation. The ticks from the control chamber were sprayed with distilled water. Acaricide sprays were carried out on days D + 7, D + 11 and D + 16 after infestation, when the ticks were predominantly in the phases of non-engorged nymph, engorged nymph, and young adults, respectively. These tick stages were chosen due to the results obtained in experiment 1 and the knowledge available in the literature [1,2,3,4,6].

The acaricide spraying consisted in directing 10 jets through the interior of a 5 cm diameter and 8 cm long tube, with each jet corresponding to a volume of 0.65 mL. The tube was positioned in place to prevent drift that could contaminate ticks from another group. The spray equipment used was a 0.5 L manual trigger spray. The internal area of the chambers was photographed daily from D + 7 after infestation using a digital camera (PowerShot G15 digital camera—Canon Inc., Tokyo, Japan). The images were analyzed for the quantification of the individuals before and after the treatments. At the time of the inspection, all fully fixed or detached engorged females were collected. The chambers were removed on D + 21 and the remaining individuals were collected, quantified, and classified as dead or alive.

### 2.4. Experiment 3: Spray Treatment of Tick Females in Feeding Chambers

Two lactating cows were chosen and had one feeding chamber fixed in the region of the perineum. The chamber was produced with the same material as mentioned above, in a triangular shape with each side measuring 20 cm and a circular cover 12 cm in diameter. The interior allowed access to an area of skin of eight cm in diameter. The chambers were fixed with the apex of the triangle directed downwards, also by means of contact glue and, in this case, with three subcutaneous stitches. The animals were anesthetized according to the same protocol described above. After that, tick infestations were conducted with 1200 larvae 45 days old.

On the day that the first engorged female detachment was observed, all fully engorged females were removed from the chamber, including those that were still attached. Subsequently, the remaining ticks, predominantly males and partially engorged females, were sprayed with acaricide. Each chamber was treated with 20 jets of a 0.5 L manual trigger spray in which each jet corresponded to 0.65 mL. The chambers were inspected daily and the fully engorged females were collected upon detachment.

Half of the fully engorged females collected before and after the acaricide treatment were submitted to AIT [8] and then maintained in a BOD incubator at 26 ± 1 °C and 85 ± 5% RH. The other half of the engorged females were incubated directly under the same conditions. Then, four groups were formed:

NT: made up of engorged females obtained from non-treated partially engorged females that were not submitted to AIT;

NT-AIT: also made up of engorged females obtained from non-treated partially engorged females, but submitted to AIT;

T: made up of engorged females obtained from partially engorged females that survived the acaricide treatment and were not submitted to AIT;

T-AIT: also made up of engorged females obtained from partially engorged females that survived the acaricide treatment but submitted to AIT.

All female oviposition was evaluated for egg viability on a stereoscopic microscope, weighed, packaged in syringes with the upper-end cut sealed with hydrophilic cotton, and incubated under the same conditions described above. Females with no oviposition or with dehydrated appearance and disaggregated eggs were considered not viable, whereas those with even partially embryonic oviposition were considered viable. After a further 18 days, larvae hatchability was performed using a stereoscopic microscope and a visual evaluation of the percentage of eggs that hatched in relation to the total. The egg production index (EPI) was calculated using the formula EPI = Weight of the egg mass (g) × 100/Initial weight of the female, according to Bennett [15].

### 2.5. Statistical Analysis

For the first experiment, the counting data was analyzed by the Kolmogorov–Smirnov normality test and the non-parametric Kruskal–Wallis test. Mortality values were evaluated by Kolmogorov–Smirnov normality test and means of analysis of variance (ANOVA), considering the level of rejection of the null hypothesis *p* < 0.05. Mortality data were also used for graph construction, and the behavior of the curves was assessed by chi-square goodness-of-fit test [16].

For the second experiment, the observed values were used to calculate mortality values and to apply the two-sample z-test for proportions [16]. The significance level was *p* < 0.05.

For the third experiment, all data was analyzed by the Kolmogorov–Smirnov normality test. Data with normal distribution were submitted to analysis of variance (ANOVA) followed by the Tukey test. Data with non-normal distribution were submitted to Kruskal–Wallis non-parametric analysis followed by Dunn’s post-test. The significance level was *p* < 0.05. The relationship between the viable and non-viable engorged female’s treatments was assessed using the chi-square test [16].

## 3. Results

### 3.1. Experiment 1: Acaricide Efficacy in Naturally Infested Cattle

**Figure 2:**
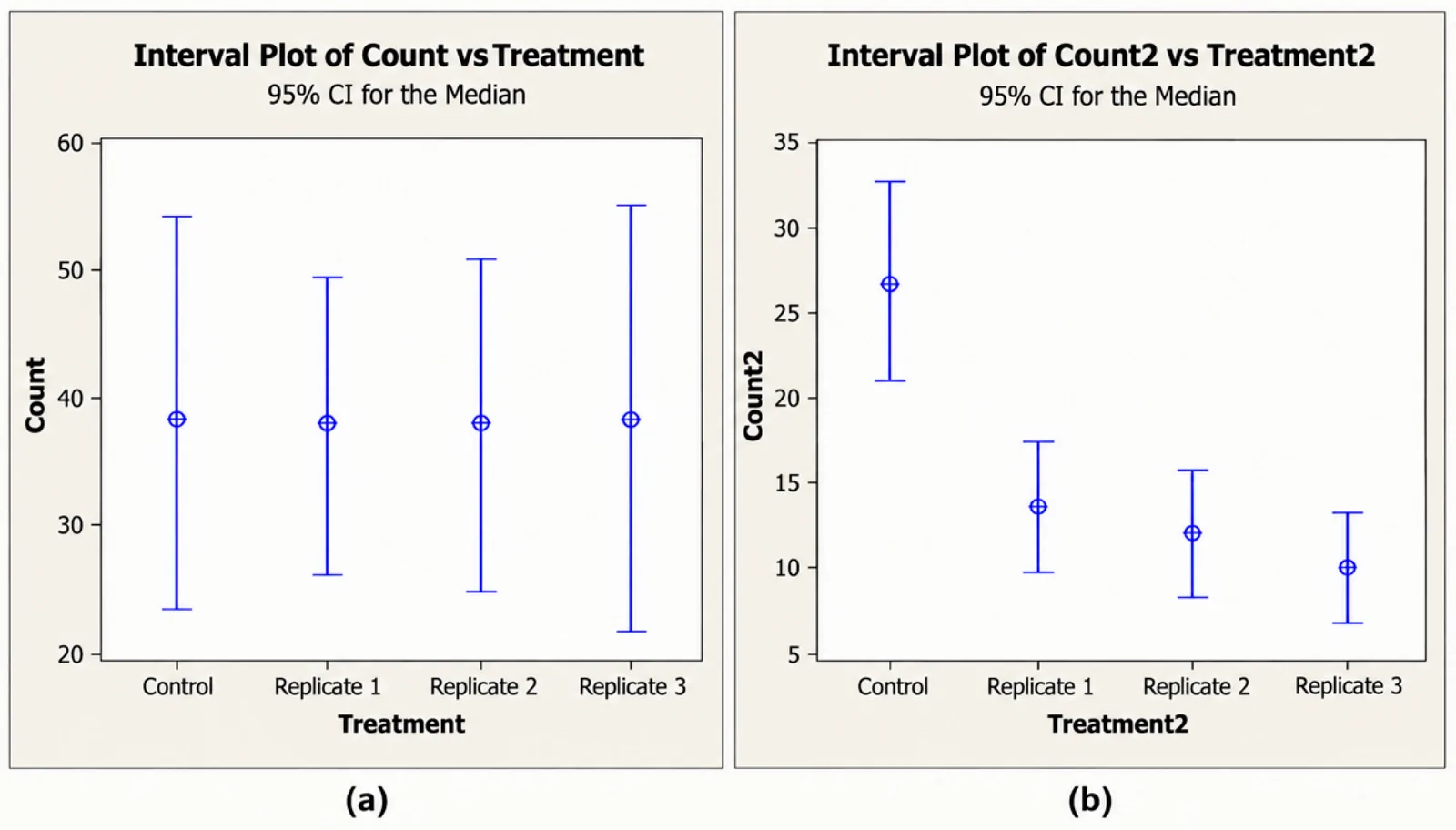
Tick counts before and after acaricide treatment. (**a**) Tick counts recorded in the experimental groups before acaricide administration, showing no significant differences among the groups (Kruskal–Wallis test, H = 0.87, df = 3, *p* = 0.832). (**b**) Tick counts after acaricide treatment. Tick counts differed significantly among the experimental groups (Kruskal–Wallis test, H = 41.44, df = 3, *p* < 0.000). The untreated control group exhibited significantly higher tick counts than all treated groups, whereas no significant differences were detected among the three treatment replicates. Data are presented as means with 95% confidence intervals.

Tick counts for the evaluated animal groups showed no differences prior to the administration of the acaricide treatment (Figure 2a). Following treatment, the treated groups did not differ from one another, yet all differed from the control group (Figure 2b). In the adult immersion test performed prior to this experiment, the acaricide used showed 100% efficacy [8].

The minimum and maximum mortality values observed on days D + 1, D + 4, D + 8, D + 11, D + 14, D + 17, D + 19, and D + 21 after acaricidal treatment were 14.0 and 44.5%; 70.0 and 80.7%; 21.2 and 31.4%; 0.0 and 1.7%; 47.9 and 67.1%; 80.1 and 85.3%; 88.7 and 93.0%; and 89.4 and 95.7%, respectively. The lowest efficacy levels were observed on days D + 1, D + 8, and D + 11 post-treatments; these did not differ from one another but differed from the other evaluation days. Day D + 14 did not differ from D + 4 post-treatment but differed from the other evaluation days. Days D + 17, D + 19, and D + 21 presented the highest efficacies and did not differ from each other or from D + 4, but they differed from the other dates (Table 1). The acaricidal efficacy of cypermethrin 0.02% and chlorpyrifos 0.03% emulsion showed 100% efficacy on AIT, whereas the efficacy against the same population under field conditions showed an average efficacy of 56.2%.

**Figure 3:**
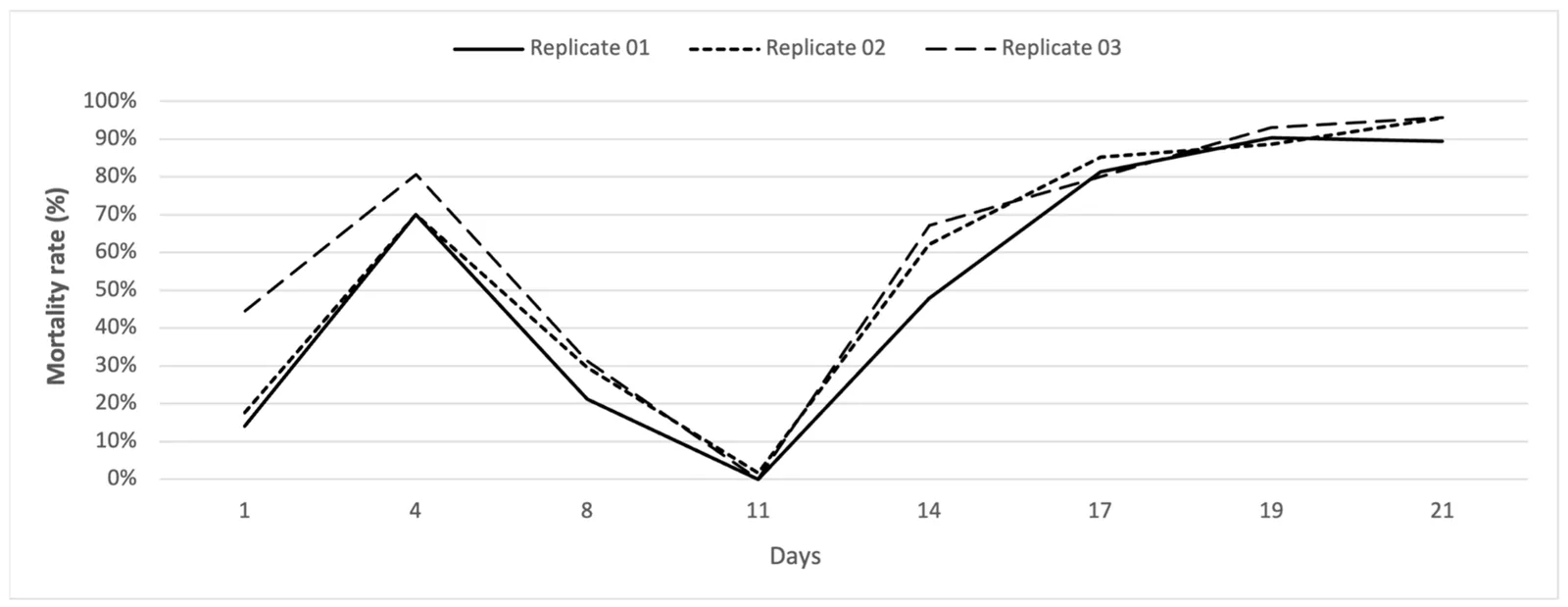
Mortality of *R. microplus* after acaricide spraying on different assessment days.

The observed mortality curves did not differ in shape; the probability of this occurring by chance was less than 5% (*p* < 0.05) according to the chi-square goodness-of-fit test (Figure 3).

### 3.2. Experiment 2: Acaricide Efficacy in Feed Chambers

In the adult immersion test performed prior to this experiment, the acaricide used showed 100% efficacy [8]. After the chambers were removed, a total of 118 males and 221 females (1.87 females/male) were counted, including seven early-stage females, 59 partially engorged females, and 155 engorged females. In addition, two metanymphae, five early-stage females, five early-stage males, and one partially engorged female were dead at the time they were collected.

**Figure 4:**
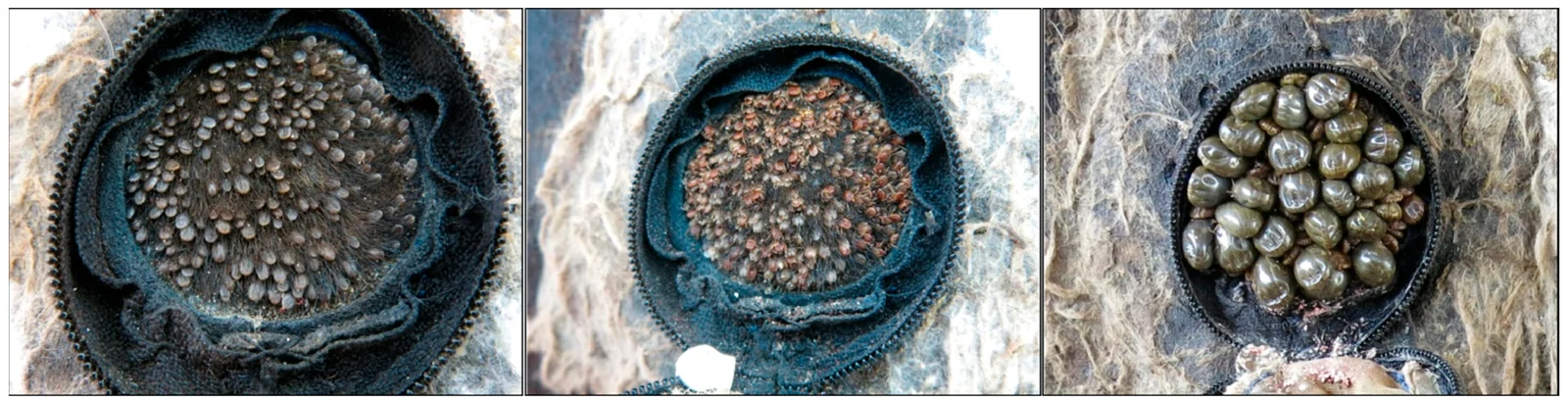
Sequence of photographs of *R. microplus* parasitic stages on treatment day D + 11, D + 16 and D + 20, from left to right, after experimental infestation with larvae and acaricidal treatment with 0.02% cypermethrin + 0.03% chlorpyrifos emulsion performed on day 11, when engorged nymphs predominated.

**Figure 5:**
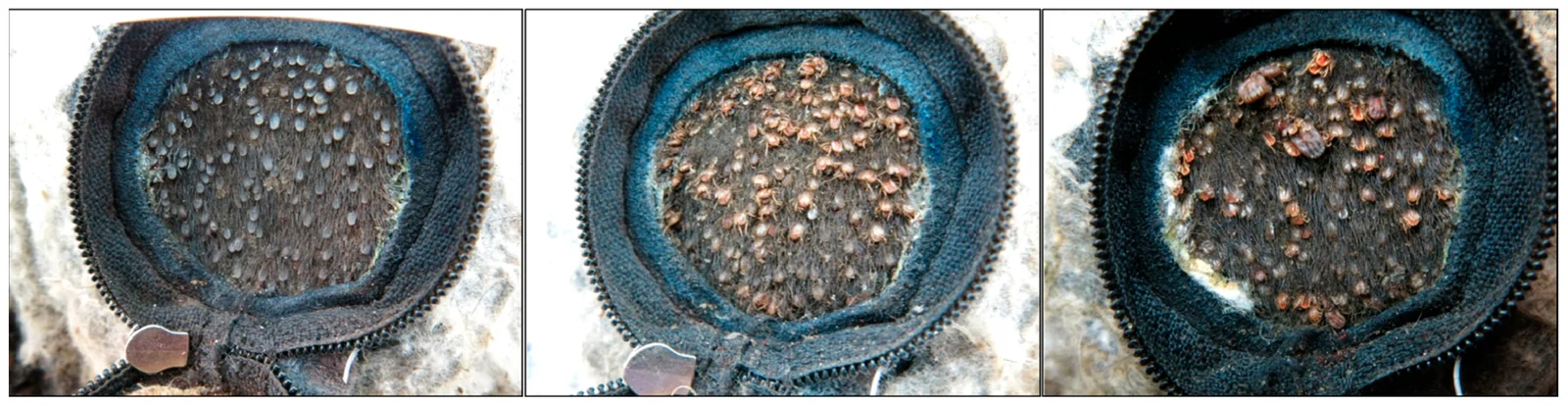
Sequence of photographs of *R. microplus* parasitic stages on days D + 11, D + 16, and D + 20, from left to right, after experimental infestation with larvae and acaricidal treatment with 0.02% cypermethrin + 0.03% chlorpyrifos emulsion on day 16, when young adults predominated.

The groups treated during the unfed nymph and young adult stages—on days D + 7 and D + 16, respectively—exhibited the highest mortality values and did not differ significantly from each other. In contrast, the engorged nymph group and the control group showed the lowest rates, differing from each other and from the other groups (Table 2). In the images used to quantify the mortality of the evaluated parasitic stages, the difference is clearly visible, particularly regarding engorged nymphs and young adults (Figure 4 and Figure 5).

### 3.3. Experiment 3: Biological Parameters of Engorged Tick Females After Spray Treatment at Partially Engorged Stage

In the adult immersion test performed prior to this experiment, the acaricide used showed 85.8% efficacy [8]. Before acaricide spray treatment, a total of 16 engorged females were collected.

The NT group showed an average engorged female weight of 258 mg, an average oviposition weight of 149 mg, hatchability of 99.5% and egg conversion index of 57.7%.

The NT-AIT group showed an average engorged female weight of 261 mg, an average oviposition weight of 43 mg, hatchability of 47.5%, and egg production index of 16.4%. In this case, product efficacy was 86.5%.

For the T group, 70 engorged females were collected. All of them laid eggs that showed 95% hatchability and the egg production index was 55.6%.

For the T-AIT group, 72 engorged females were collected and, after the immersion test, 17 females laid eggs. The average egg mass weighed 27 mg, the hatching rate was 15%, and the egg production index was 9.0%. In this case, the product’s efficacy was 97.7%.

No difference in the mean weight of engorged females was observed between the T and T-AIT groups (Table 3). However, differences were observed by analysis of variance (ANOVA) for oviposition weight (*p* < 0.000) and hatchability (*p* < 0.000), and by the Kruskal–Wallis test for egg conversion index (*p* < 0.004) and reproductive efficiency (*p* < 0.004) (Table 4). Also, the number of viable engorged females in the T group (69) was significantly higher (*p* < 0.05) than the 17 observed in the T-AIT group.

## 4. Discussion

The main finding of this study is that acaricide susceptibility in *R. microplus* varies according to the parasitic developmental stage and, particularly, to engorgement status. This pattern was consistently observed across the three experimental approaches, with lower susceptibility associated with engorged nymphs, metanymphs, and partially or fully engorged females, whereas non-engorged nymphs and young adults were more susceptible. These findings indicate that developmental stage should be considered an important source of variation when interpreting acaricide efficacy under field conditions.

Many early reports concerning *R. microplus* parasitic stages that appeared to survive acaricide spraying, unlike those eliminated or reduced, were based on observations of the detachment of engorged females [1,2,3,4]. Larval stages occur between the first and fifth days after the onset of blood feeding; nymphal stages between the sixth and fifteenth days; and adult stages are observed from the sixteenth day onwards. The modal day for the detachment of engorged females is reported to be the twenty-second day [17]. Given the characteristic developmental timeframe for each stage, it is reasonable to assume that the detachment of engorged females at specific times post-treatment is linked to individuals at specific stages that completed the parasitic cycle within a similar period. So, female counts during the first three weeks after acaricide spray treatment would also reflect surviving individuals, and each assessment date would predominantly correspond to a given stage [13].

Counts performed on days D + 1, D + 4, D + 8, D + 11, D + 14, D + 17, D + 19, and D + 21, as in this study, would therefore represent predominantly and, respectively, individuals in the partially engorged female stage; young adults; males and females; metanymphs; partially engorged nymphs; non-engorged nymphs just after molting; engorged larvae; partially engorged larvae; and non-engorged larvae that were on the host on the day of spraying [17,18,19]. Consequently, the mortality calculated for each date would represent the resistance/susceptibility index for the predominantly corresponding stages.

Some limitations of the field experiment should be considered when interpreting these results. Because the hosts were maintained on infested pastures, natural infestation may have affected the later count dates; larvae attaching up to three days after treatment could have been counted as partially engorged females between D + 19 and D + 21, potentially underestimating mortality during this period. Other possible sources of interference include intermediate levels of intoxication capable of prolonging the parasitic period, and residual acaricide activity causing mortality after ticks had transitioned to a subsequent developmental stage. Nevertheless, observations from Experiments 2 and 3 indicated that surviving individuals completed their life cycle within the expected timeframe, and no evident residual mortality was observed after developmental transitions. Thus, although these potential sources of interference cannot be completely excluded, their impact under the experimental conditions appears to have been limited.

Knowledge regarding the biology of the parasitic stages of *R. microplus* has long been established and demonstrates the existence of a relationship between count date and developmental stage after the infestation date [17,18,19]. The observations from this study indicate that this relationship can also be considered after acaricide spray treatment. This could enable an increase in the amount of information obtained from stable and field tests and may improve the understanding of treatment effects.

Most authors who reported the possibility that some parasitic stages could be more resistant than others attributed this ability to partially engorged females and metanymphae [1,2,3,4], observing that engorged females showed similar drop-off patterns in more than 30 field tests evaluating the efficacy of different organophosphates and arsenic. After spray treatments, several females still completed engorgement and detached, mainly up to three days later. But a “second wave” of detachment, as the authors termed it, between nine and 12 days was attributed to individuals that survived treatment while in the metanymphal stage.

In the present study, the mortality curves at field test, in addition to being consistent with each other (*p* < 0.000), showed a shape with two waves of detachment like that reported by Roulston et al. [4]; however, they appeared as a “depression,” since mortality data were plotted, which show the inverse pattern. In several studies evaluating acaricide efficacy [20,21,22,23], it was also possible to observe lower efficacies in the data for the dates corresponding to engorged nymphs, metanymphae, and partially engorged females.

In the second experiment of this study, engorged nymphs were more resistant than non-engorged nymphs and young adults, and the mortality values were close to those observed in the field tests. Similarly, in the third test, it was possible to demonstrate low mortality values for partially engorged females, also with values close to those observed in the field tests. The findings from the three experiments were consistent with each other and with the proposed hypothesis. The results obtained agree with the reports [1,2,3,4] and with the study that previously demonstrated it [6]. It is important to emphasize, however, that the data refer to a specific population exposed to treatments with apyrethroid-organophosphate combination. Souza [6], who worked under laboratory conditions with a resistant strain, observed mortality values close to zero for metanymphs and partially and fully engorged females after immersion in ethion and amitraz, but not arsenic.

In the study by [5]—the only one to reach a conclusion contrary to that of other reports involving organophosphates—the authors observed mortality values approaching 100% in nymphal instars and partially engorged females. Although the nymphs completed the molt and died as young adults, the use of a susceptible strain likely prevented the observation of greater resistance at that developmental stage. Differences in susceptibility may be more clearly discernible in populations with intermediate levels of resistance than in highly susceptible or resistant strains.

In the field trials, the lowest mortality values were recorded on days 1, 8, and 11, coinciding respectively with the stages of fully and partially engorged females, metanymphs, and engorged nymphs. Day 11 is noteworthy, because the observed mortality was null and significantly lower than the others. In addition, assessment on the second experiment demonstrated the high resistance of engorged nymphs when they had not yet started the molting process. Thus, the hypothesis of a double epicuticle layer resulting from the molting process loses weight [4]. If the double-layered epicuticule were the primary reason for the resistance, one would expect high levels of resistance to be relatively short-lived and most pronounced during the final days of the nymphal stage, and perhaps not as noticeable in semi-engorged females—which differs from what was observed.

Another interesting issue is the apparent tendency toward increased resistance as the level of engorgement increases and development proceeds (Table 1). Larvae were more susceptible than nymphs, non-engorged nymphs were more susceptible than engorged nymphs and metanymphs, and young adults were more susceptible than partially and fully engorged females. However, differences between larval stages were not observed either between non-engorged nymphs or early male and female stages. A few hypotheses—which are not mutually exclusive—have been proposed and discussed as possible ways to help explain the observed differences, as follows.

**Hypothesis** **1** **(H1).** *Rapid engorgement at the end of the feeding period of each stage causes a marked increase in body volume and a drastic reduction in the surface area-to-volume ratio. The higher an organism’s (or tissue’s) S/V ratio, the greater the contact area with the external environment per unit of mass—and, consequently, the higher the absorption rate, speed of uptake, and risk of toxicity [24]. Furthermore, the surface area-to-volume (S/V) ratio determines the rate of contaminant absorption in the context of pharmacokinetic allometry. It demonstrates how a drug’s volume of distribution and half-life vary directly with body mass [25].*

In the case of tick larvae, engorgement results in an almost eightfold weight increase, rising from approximately 0.031 mg to 0.236 mg. For nymphs, engorgement leads to a nearly tenfold weight increase, ranging from 0.200 mg to a maximum of 1.930 mg. In the case of females, engorgement causes a weight increase of nearly 158-fold, rising from approximately 1.900 mg to reach an engorged female weight of 300 mg. From the larval to the engorged female stages, the tick’s weight increases approximately 10,000-fold. These data were calculated based on collected specimen measurements and the literature by [17,19]. Given that topical acaricides are absorbed primarily through the integument, the marked reduction in the surface area-to-volume ratio should contribute to decreasing the relative amount of active ingredient absorbed.

**Hypothesis** **2** **(H2).** *During blood feeding, ticks ingest blood, return plasma and saliva to the host, and excrete. The quantity and composition of saliva also show marked variation, particularly during the final moments of engorgement [26]. Such behaviors cease from the moment engorged females detach and during the molting process. Both excreta and saliva could serve as vehicles for the elimination of the active ingredient.*

This form of clearance depends on the excretory and digestive systems and is related to the mechanisms regulating hydroelectrolytic balance in most living organisms [26,27]. In the third experiment, it was possible to observe high viability of partially engorged females that completed blood feeding after acaricidal treatment. Those incubated without undergoing additional treatment exhibited normal bionomics, like that of unexposed ticks. By contrast, those subjected to a new treatment after detachment showed high mortality and low reproductive efficiency. These marked differences indicate that some specific conditions contribute significantly to the greater resistance observed during engorgement than after detachment. The observation of large amounts of excreta during the engorgement periods drew attention and motivated the formulation of this hypothesis. Although Souza [6] is the only study that evaluated semi-engorged females removed from the host and subjected to immersion in ethion and amitraz, it is important to note that they observed near-zero susceptibility.

Tegument-related issues cannot be ruled out, although it remains uncertain how these processes, characteristic of the physiology of parasitic stages, may contribute to greater or lesser susceptibility. During molting, integument layers are lost through digestion and/or synthesis cessation, while the layers of the new tegument are still being synthesized [26]. The tegument thickening that occurs during the feeding period and precedes rapid engorgement could represent a more efficient barrier against the penetration of contact-acting active ingredients, since the thickness of a given membrane affects the absorption rate per unit of body mass—which is directly proportional to the S/V ratio, as discussed in Hypothesis 1 [24]. During this period, the basal cells of the endothelium increase in number and volume and assume a folded shape, preparing the structure to withstand rapid distension [26]. Considering the obtained results, this aspect would help explain the resistance observed in partially engorged females. However, in attached fully engorged individuals, such as engorged nymphs and engorged females, the tegument is already distended. It is important, therefore, to consider the possibility that several factors contribute.

Finally, another important point concerns the differences frequently observed between laboratory efficacy tests and treatments conducted under field conditions. As early as 1968, Roulston et al. [4] drew attention to the reduced efficacy of acaricidal compounds under field conditions. In the present study, while acaricide efficacy reached 100% under laboratory conditions (AIT), spray treatment under field conditions resulted in an average efficacy of only 56.2%.

From a practical perspective, the stage-dependent differences in susceptibility observed here may help explain this discrepancy. Conventional laboratory protocols generally assess the mortality and reproductive performance of engorged females or the mortality of larvae after controlled contact or immersion tests [8,28], whereas field efficacy tests simultaneously expose ticks at different parasitic developmental and engorgement stages [13]. Field outcomes are also influenced by environmental and host-related factors and by differences in the mode of acaricide exposure [8,13,28]. Thus, laboratory and field methodologies differ not only in how ticks are exposed to acaricide, but also in the developmental and physiological conditions of the individuals being evaluated [26]. Our findings indicate that stage-dependent susceptibility represents an additional source of variation that may contribute to the lower efficacy frequently observed under field conditions and should therefore be considered when interpreting laboratory susceptibility assays and predicting treatment performance. Further studies are needed to clarify the physiological mechanisms underlying these differences and to determine their contribution to discrepancies between laboratory and field efficacy.

## 5. Conclusions

The present study provides consistent evidence, from field, in vivo, and AIT experiments, that the susceptibility of *R. microplus* to spray acaricides varies markedly among parasitic developmental stages. Lower susceptibility was consistently associated with metanymphs, engorged nymphs, and partially or fully engorged females, whereas non-engorged larvae, non-engorged nymphs, and young adults were more susceptible. These findings indicate that the reduced susceptibility traditionally attributed to the double epicuticle during molting cannot fully explain the observed pattern. Instead, physiological changes associated with feeding and development, including progressive engorgement, are likely to play an important role. This stage-dependent variation in susceptibility provides a plausible explanation for the recurrent discrepancies between laboratory susceptibility assays and field efficacy trials. Recognizing these differences is essential for improving the interpretation of acaricide efficacy studies, refining laboratory protocols, and developing control strategies that better predict field performance. Further studies are needed to identify the physiological mechanisms underlying these differences and to determine whether the observed pattern is consistent across different acaricide classes and tick populations.

## Figures and Tables

**Table 1:** Tick mortality values at different days after acaricide spray treatment with 0.02% cypermethrin + 0.03% chlorpyrifos emulsion.

	Days After Treatment								Total
	D + 1	D + 4	D + 8	D + 11	D + 14	D + 17	D + 19	D + 21	
Average mortality	25.4% ^a^	74.6% ^bc^	27.4% ^a^	0.0% ^a^	59.0% ^b^	82.3% ^c^	90.7% ^c^	93.6% ^c^	56.2%

Different letters within the same row indicate a significant difference by one-way analysis of variance (ANOVA), *p* < 0.000.

**Table 2:** Mortality at different parasitic stages of *Rhipicephalus microplus* after “in vivo” acaricide spray treatment with 0.02% cypermethrin + 0.03% chlorpyrifos emulsion.

Groups	Treated Ticks	Surviving Ticks	Dead Ticks	Mortality *
Control	115	106	9	7.8% ^c^
D + 7	192	33	159	83.2% ^a^
D + 11	260	184	76	29.2% ^b^
D + 16	168	17	151	89.9% ^a^

* Different letters within the same column indicate a significant difference according to the two-sample z-test for proportions (*p* < 0.05).

**Table 3:** Weight of recovered engorged females after “in vivo” acaricide spray treatment at the moment they reached the partially engorged female stage, divided into groups not submitted (T) and submitted (T-AIT) to the adult immersion test.

Groups	Engorged Females		
	Collected	Total Weight (g)	Average Weight (g) *
T	70	20.481	0.293 ^a^
T-AIT	72	21.515	0.299 ^a^

* Identical letters within the same column indicate that there was no significant difference (*p* > 0,05), as assessed by statistical testing.

**Table 4:** Oviposition average weight and hatchability from engorged females recovered after “in vivo” acaricide spray treatment at the moment they reached the partially engorged female stage, divided into groups not submitted (T) and submitted (T-AIT) to the adult immersion test.

Groups	Oviposition	
	Average Weight (g) *	Hatchability *
T	0.163 ^a^	95% ^a^
T-AIT	0.027 ^b^	15% ^b^

* Different letters within the same column indicate a significant difference, as assessed by statistical testing 15%.

## Data Availability

The data supporting the findings of this study are available from the corresponding author upon reasonable request.

## Abbreviations

The following abbreviations are used in this manuscript:

AIT	Adult immersion test
BOD	Biochemical Oxygen Demand
EPAMIG	Empresa Agropecuária de Minas Gerais
REI	Reproductive efficiency index
UFMG	Universidade Federal de Minas Gerais
